# 2-(4-Chloro­phen­yl)acetamide

**DOI:** 10.1107/S1600536811046836

**Published:** 2011-11-12

**Authors:** Dong-Sheng Ma, Pei-Jiang Liu, Shuai Zhang, Guang-Feng Hou

**Affiliations:** a College of Chemistry and Materials Science, Heilongjiang University, Harbin 150080, People’s Republic of China

## Abstract

In the title compound, C_8_H_8_ClNO, the acetamide group is twisted out the benzene plane with a dihedral angle of 83.08 (1)°. In the crystal, mol­ecules are linked by N—H⋯O hydrogen bonds, forming layers parallel to the *ab* plane.

## Related literature

For details of the nitrile hydrolysis of the same substrate (4-chlorobenzonitrile) by another method, see: Moorthy & Singhal (2005 ▶).
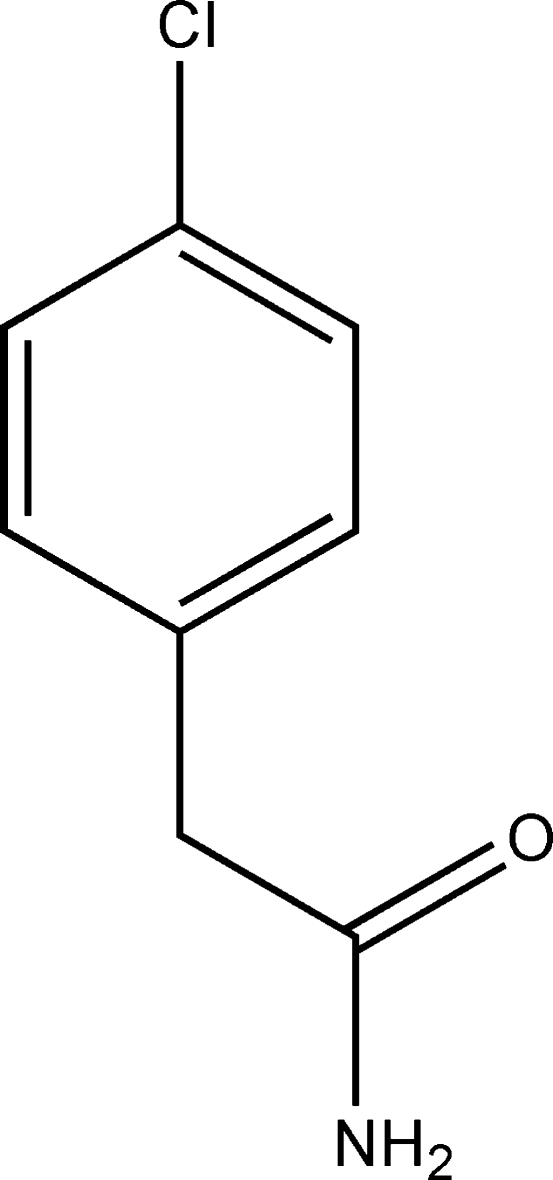


## Experimental

### 

#### Crystal data

C_8_H_8_ClNO
*M* *_r_* = 169.60
Orthorhombic, 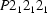

*a* = 4.917 (2) Å
*b* = 6.033 (4) Å
*c* = 26.680 (12) Å
*V* = 791.5 (7) Å^3^
*Z* = 4
Mo *K*α radiation
μ = 0.42 mm^−1^
*T* = 293 K
0.29 × 0.22 × 0.07 mm

#### Data collection

Rigaku R-AXIS RAPID diffractometer
Absorption correction: multi-scan (*ABSCOR*; Higashi, 1995 ▶) *T*_min_ = 0.887, *T*_max_ = 0.970
7733 measured reflections
1807 independent reflections
1451 reflections with *I* > 2σ(*I*)
*R*_int_ = 0.041

#### Refinement

*R*[*F* ^2^ > 2σ(*F* ^2^)] = 0.037
*wR*(*F* ^2^) = 0.083
*S* = 1.05
1807 reflections
108 parameters
2 restraints
H atoms treated by a mixture of independent and constrained refinement
Δρ_max_ = 0.17 e Å^−3^
Δρ_min_ = −0.17 e Å^−3^
Absolute structure: Flack (1983 ▶), 704 Friedel pairs
Flack parameter: −0.12 (8)

### 

Data collection: *RAPID-AUTO* (Rigaku, 1998 ▶); cell refinement: *RAPID-AUTO*; data reduction: *CrystalClear* (Rigaku/MSC, 2002 ▶); program(s) used to solve structure: *SHELXS97* (Sheldrick, 2008 ▶); program(s) used to refine structure: *SHELXL97* (Sheldrick, 2008 ▶); molecular graphics: *SHELXTL* (Sheldrick, 2008 ▶); software used to prepare material for publication: *SHELXL97*.

## Supplementary Material

Crystal structure: contains datablock(s) I, global. DOI: 10.1107/S1600536811046836/cv5191sup1.cif

Structure factors: contains datablock(s) I. DOI: 10.1107/S1600536811046836/cv5191Isup2.hkl

Supplementary material file. DOI: 10.1107/S1600536811046836/cv5191Isup3.cml

Additional supplementary materials:  crystallographic information; 3D view; checkCIF report

## Figures and Tables

**Table 1:** Hydrogen-bond geometry (Å, °)

*D*—H⋯*A*	*D*—H	H⋯*A*	*D*⋯*A*	*D*—H⋯*A*
N1—H11⋯O1^i^	0.88 (1)	2.05 (1)	2.911 (2)	165 (2)
N1—H12⋯O1^ii^	0.89 (1)	2.22 (1)	3.064 (3)	157 (2)

Symmetry codes: (i) 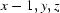; (ii) 
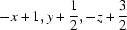
.

**Fig. 1.**
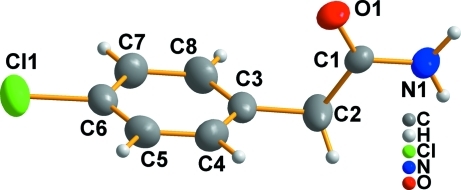
The molecular structure of the title compound, showing displacement ellipsoids at the 50% probability level.

**Fig. 2.**
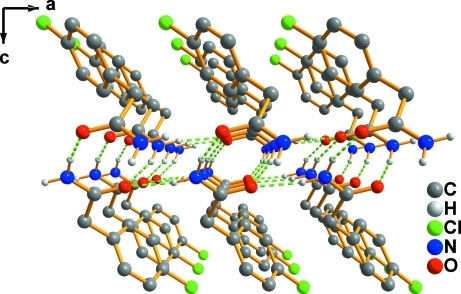
A portion of the crystal packing, showing a two-dimensional structure formed by N—H···O hydrogen bonds (dashed lines).

## supplementary crystallographic information

### Comment

The title compound is formed by hydrolysis of appropriate nitriles (Moorthy *et al.*, 2005), while the final product of hydrolysis of nitriles should be carboxylic acid. In this paper, we report the synthesis and the crystal structure of the title compound prepared from 4-cyanobenzylchloride under solvothermal condition.

In the title molecule (Fig.1), the acetamide group is twisted out the benzene plane with a dihedral angle of 83.08 (1) °. In the crystal packing, the molecules are linked by N—H···O hydrogen bonds to form layers parallel to *ab* plane (Fig. 2, Table 1).

### Experimental

A mixture of NaN_3_ (0.39 g, 6 mmol), CuCl_2_^.^2H_2_O (0.684 g, 4 mmol), and 4-cyanobenzylchloride (0.606 g, 4 mmol) was sealed in a 15 ml teflon-lined reactor and heated in an oven at 150 ° C for 72 hrs and slowly cooled to room temperature. The resulting mixture was washed with water, and pale yellow blocklike crystals were collected (yeild 31%).

### Refinement

N-bound H atoms were located in a differece Fourier map and refined with restraint of N—H = 0.89 (1) Å. C-bound H atoms were placed in calculated positions and treated as riding on their parent atoms, with C—H = 0.93 Å (aromatic); C—H = 0.97 Å (methylene), and with *U*_iso_(H) = 1.2*U*_eq_(C).

### Crystal data

**Table:**

C_8_H_8_ClNO	*F*(000) = 352
*M**_r_* = 169.60	*D*_x_ = 1.423 Mg m^−^^3^
Orthorhombic, *P*2_1_2_1_2_1_	Mo *K*α radiation, λ = 0.71073 Å
Hall symbol: P 2ac 2ab	Cell parameters from 5994 reflections
*a* = 4.917 (2) Å	θ = 3.1–27.4°
*b* = 6.033 (4) Å	µ = 0.42 mm^−^^1^
*c* = 26.680 (12) Å	*T* = 293 K
*V* = 791.5 (7) Å^3^	Block, colorless
*Z* = 4	0.29 × 0.22 × 0.07 mm

### Data collection

**Table:**

Rigaku R-AXIS RAPID diffractometer	1807 independent reflections
Radiation source: fine-focus sealed tube	1451 reflections with *I* > 2σ(*I*)
graphite	*R*_int_ = 0.041
ω scan	θ_max_ = 27.4°, θ_min_ = 3.1°
Absorption correction: multi-scan (*ABSCOR*; Higashi, 1995)	*h* = −6→6
*T*_min_ = 0.887, *T*_max_ = 0.970	*k* = −7→7
7733 measured reflections	*l* = −34→33

### Refinement

**Table:**

Refinement on *F*^2^	Secondary atom site location: difference Fourier map
Least-squares matrix: full	Hydrogen site location: inferred from neighbouring sites
*R*[*F*^2^ > 2σ(*F*^2^)] = 0.037	H atoms treated by a mixture of independent and constrained refinement
*wR*(*F*^2^) = 0.083	*w* = 1/[σ^2^(*F*_o_^2^) + (0.036*P*)^2^ + 0.1017*P*] where *P* = (*F*_o_^2^ + 2*F*_c_^2^)/3
*S* = 1.05	(Δ/σ)_max_ < 0.001
1807 reflections	Δρ_max_ = 0.17 e Å^−^^3^
108 parameters	Δρ_min_ = −0.17 e Å^−^^3^
2 restraints	Absolute structure: Flack (1983), 704 Friedel pairs
Primary atom site location: structure-invariant direct methods	Flack parameter: −0.12 (8)

### Special details

**Table:**

Geometry. All esds (except the esd in the dihedral angle between two l.s. planes) are estimated using the full covariance matrix. The cell esds are taken into account individually in the estimation of esds in distances, angles and torsion angles; correlations between esds in cell parameters are only used when they are defined by crystal symmetry. An approximate (isotropic) treatment of cell esds is used for estimating esds involving l.s. planes.
Refinement. Refinement of F^2^ against ALL reflections. The weighted R-factor wR and goodness of fit S are based on F^2^, conventional R-factors R are based on F, with F set to zero for negative F^2^. The threshold expression of F^2^ > 2sigma(F^2^) is used only for calculating R-factors(gt) etc. and is not relevant to the choice of reflections for refinement. R-factors based on F^2^ are statistically about twice as large as those based on F, and R- factors based on ALL data will be even larger.

### Fractional atomic coordinates and isotropic or equivalent isotropic displacement parameters (Å^2^)

**Table:**

	*x*	*y*	*z*	*U*_iso_*/*U*_eq_	
C1	0.3632 (4)	0.5073 (3)	0.79843 (7)	0.0335 (4)	
C2	0.2527 (4)	0.3677 (5)	0.84087 (10)	0.0603 (7)	
H2A	0.1560	0.4638	0.8639	0.072*	
H2B	0.1222	0.2633	0.8272	0.072*	
C3	0.4640 (4)	0.2408 (4)	0.86973 (8)	0.0429 (5)	
C4	0.5575 (5)	0.0374 (4)	0.85279 (8)	0.0487 (6)	
H4	0.4907	−0.0195	0.8228	0.058*	
C5	0.7475 (5)	−0.0822 (3)	0.87944 (8)	0.0451 (5)	
H5	0.8079	−0.2185	0.8676	0.054*	
C6	0.8470 (4)	0.0017 (4)	0.92375 (8)	0.0410 (5)	
C7	0.7620 (5)	0.2049 (4)	0.94146 (8)	0.0472 (6)	
H7	0.8324	0.2622	0.9711	0.057*	
C8	0.5697 (5)	0.3221 (4)	0.91430 (8)	0.0487 (5)	
H8	0.5100	0.4586	0.9262	0.058*	
Cl1	1.08553 (13)	−0.14906 (11)	0.95804 (2)	0.0605 (2)	
N1	0.1788 (3)	0.6234 (4)	0.77353 (7)	0.0441 (4)	
H11	0.004 (2)	0.613 (4)	0.7811 (8)	0.046 (6)*	
H12	0.225 (5)	0.714 (4)	0.7485 (7)	0.064 (8)*	
O1	0.6073 (3)	0.5149 (3)	0.78808 (5)	0.0435 (4)	

### Atomic displacement parameters (Å^2^)

**Table:**

	*U*^11^	*U*^22^	*U*^33^	*U*^12^	*U*^13^	*U*^23^
C1	0.0263 (9)	0.0376 (11)	0.0367 (10)	−0.0005 (9)	0.0007 (8)	−0.0027 (8)
C2	0.0291 (11)	0.0819 (19)	0.0699 (15)	0.0050 (12)	0.0050 (11)	0.0347 (15)
C3	0.0310 (11)	0.0528 (13)	0.0449 (11)	0.0000 (9)	0.0035 (9)	0.0145 (9)
C4	0.0493 (13)	0.0554 (14)	0.0416 (11)	−0.0093 (12)	−0.0077 (11)	−0.0001 (10)
C5	0.0511 (13)	0.0368 (12)	0.0475 (12)	0.0016 (9)	0.0072 (11)	−0.0041 (9)
C6	0.0381 (11)	0.0444 (12)	0.0406 (10)	0.0018 (10)	0.0046 (9)	0.0071 (9)
C7	0.0514 (13)	0.0514 (14)	0.0387 (11)	0.0009 (10)	−0.0027 (10)	−0.0056 (10)
C8	0.0503 (13)	0.0433 (13)	0.0525 (12)	0.0118 (12)	0.0069 (12)	−0.0003 (10)
Cl1	0.0520 (3)	0.0695 (4)	0.0598 (3)	0.0147 (3)	−0.0015 (3)	0.0194 (3)
N1	0.0243 (8)	0.0587 (12)	0.0494 (10)	−0.0006 (8)	0.0013 (8)	0.0149 (10)
O1	0.0237 (6)	0.0529 (9)	0.0538 (8)	−0.0038 (7)	0.0051 (7)	0.0046 (7)

### Geometric parameters (Å, °)

**Table:**

C1—O1	1.232 (2)	C5—C6	1.376 (3)
C1—N1	1.324 (3)	C5—H5	0.9300
C1—C2	1.512 (3)	C6—C7	1.379 (3)
C2—C3	1.503 (3)	C6—Cl1	1.744 (2)
C2—H2A	0.9700	C7—C8	1.386 (3)
C2—H2B	0.9700	C7—H7	0.9300
C3—C4	1.386 (3)	C8—H8	0.9300
C3—C8	1.387 (3)	N1—H11	0.883 (10)
C4—C5	1.378 (3)	N1—H12	0.892 (10)
C4—H4	0.9300		
			
O1—C1—N1	122.34 (19)	C6—C5—C4	119.5 (2)
O1—C1—C2	122.57 (18)	C6—C5—H5	120.3
N1—C1—C2	115.08 (17)	C4—C5—H5	120.3
C3—C2—C1	114.78 (17)	C5—C6—C7	120.9 (2)
C3—C2—H2A	108.6	C5—C6—Cl1	119.88 (18)
C1—C2—H2A	108.6	C7—C6—Cl1	119.21 (17)
C3—C2—H2B	108.6	C6—C7—C8	118.8 (2)
C1—C2—H2B	108.6	C6—C7—H7	120.6
H2A—C2—H2B	107.5	C8—C7—H7	120.6
C4—C3—C8	117.9 (2)	C7—C8—C3	121.6 (2)
C4—C3—C2	120.9 (2)	C7—C8—H8	119.2
C8—C3—C2	121.2 (2)	C3—C8—H8	119.2
C5—C4—C3	121.3 (2)	C1—N1—H11	120.9 (16)
C5—C4—H4	119.3	C1—N1—H12	121.7 (18)
C3—C4—H4	119.3	H11—N1—H12	117 (2)

### Hydrogen-bond geometry (Å, °)

**Table:**

*D*—H···*A*	*D*—H	H···*A*	*D*···*A*	*D*—H···*A*
N1—H11···O1^i^	0.88 (1)	2.05 (1)	2.911 (2)	165 (2)
N1—H12···O1^ii^	0.89 (1)	2.22 (1)	3.064 (3)	157 (2)

Symmetry codes: (i) *x*−1, *y*, *z*; (ii) −*x*+1, *y*+1/2, −*z*+3/2.
